# Medical Regulations during Battle

**Published:** 1863-05

**Authors:** William A. Hammond

**Affiliations:** Surgeon-General U. S. Army; Surgeon-General’s Office, Washington, D. C.


### MEDICAL REGULATIONS DURING BATTLE.

SURGEON-GENERAL'S OFFICE,

WASHINGTON, D. C., March 25, 1863.

In order that the wounded may receive prompt and skillful attention during and immediately after a battle, the following instructions, compiled in part from a circular issued by the Chief Medical Director of the Army of the Potomac, October 30, 1862, are published for the information and guidance of medical officers:

1. Before a battle, the Chief Medical Director, and the Medical Directors of Army Corps, will consult and co-operate with the officers of the Quartermaster's Department in making the necessary arrangements for the transportation of the wounded, and in instructing the drivers and assistants in the service of the ambulances and litters.

2. The Chief Medical Director will have the general superintendence of the whole ambulance and hospital service, and will give such orders for the removal, accommodation, and surgical treatment of the wounded as may be necessary. After a battle, he will cause the wounded to be removed to the permanent general hospitals, as soon as it is proper to do so, and no wounded man will be sent away from a field hospital without his authority.

3. As soon as practicable after a battle, the Chief Medical Director will transmit to the Surgeon-General a report of the action, describing the nature of the battle, the number engaged, the character and range of the enemy's fire, and the period and mode of removal of the wounded. He will state the number and location of the division hospitals, their organization and supplies, and also whether the wounded were promptly provided with food and blankets. He will transmit with this report a consolidated tabular statement of wounds received and operations performed. (See tabular statements, monthly report of sick and wounded.) Should deaths occur from anæsthetics, they will be reported in detail.

4. Medical Directors of army corps will apply to their commanders, on the eve of a battle, for the necessary guards, and men for fatigue duty.—These guards will be particularly careful that no stragglers be allowed about the field hospitals, using the food and comforts prepared for the wounded.

5. Previous to an engagement, each Medical Director of an army corps will detail a proper number of medical officers to remain and take care of the wounded, should a retreat be necessary. This detail he will request the corps commanders to announce in orders.

6. Medical Directors of army corps, acting under the orders of the Chief Medical Director, will exercise a general superintendence over, and direction of, the medical service of their respective corps. They will establish division field hospitals in the most convenient and secure positions, with ready access to water and fuel, and in buildings, where suitable ones can be obtained.

7. Medical Directors of corps will see that the division hospitals are properly organized and provided with the necessary medicines, instruments, stores, and furniture.

8. They will see that the ambulances which follow the troops to succor the wounded and remove them from the field, have the necessary attendants, litters, and litter bearers, so that soldiers have no excuse to leave the ranks for that object.

9. The Surgeon-in-Chief of each division will exercise general supervision, under the Medical Director of the corps, over the medical service of his division. He will see that the officers and attendants are faithful and efficient in the discharge of their duties in the hospital, and upon the field, and that the wounded are removed from the field carefully, and with despatch.

10. He will organize the division hospital as follows:

1st. A surgeon in charge; one assistant surgeon to provide food, fuel and water, and one assistant-surgeon to keep the records.

2d. Three medical officers, to constitute the operating staff of the hospital; three medical officers as assistants to each of these officers.

3d. Additional medical officers, hospital stewards, cooks and nurses of the division.

11. The surgeon in charge will have the general superintendence, and be responsible to the division surgeon for the administration of the hospital.—It will be his duty to have the hospital tents properly pitched, and when houses are used, to have them put in proper order for the reception of wounded. He is to provide the necessary medical and hospital supplies, operating tables, straw or hay for bedding, blankets, and rations.

12. The assistant-surgeons, who are under the immediate orders of the surgeon-in-charge, will aid that officer in preparing the hospital for the reception of the wounded. That duty performed, one assistant surgeon will organize and take charge of a kitchen, using for this purpose the hospital mess-chests, and the kettles, tins, etc., in the ambulances. The supplies of beef-extract and bread in the ambulances, and of extract of coffee, tea, condensed milk, and other hospital stores in the hospital supply wagons, will enable him to prepare quickly a sufficient quantity of palatable and nourishing food to meet the demands, until fresh beef and other subsistence stores can be provided. All the cooks, and such of the hospital stewards and nurses as may be necessary, will be placed under the orders of this assistant-surgeon.

13. The other assistant-surgeon will keep a complete record of every case brought to the hospital, giving the name, rank, company, and regiment; the seat and character of the injury; the treatment; the operation, if any performed; the name of the operator, and the result. This record will be transmitted by the division surgeon to the Medical Director of the corps, and by him sent to the Chief Medical Director.

14 The assistant surgeon will make out two "Tabular statements of wounded," one of which the division surgeon will transmit, within forty-eight hours after a battle, to the Chief Medical Director, and the other to the Medical Director of the corps.

15. He will also see to the proper interment of those who die, and that each grave is marked with a headboard, with the name, rank, company and regiment legibly inscribed upon it.

16. The three medical officers composing the operating staff, will be selected by the division surgeon, without regard to rank, but solely on account of their known prudence, judgment, and skill. The immediate responsibility of the performance of all important operations will rest with them. In all doubtful cases they will consult together, and a majority of them shall decide upon the expediency and character of the operation.

17. Each of these officers will have the aid of three medical officers, who, acting under his orders, will assist him in his operations.

18. The remaining medical officers of the division, except one to each regiment, will be ordered to the hospitals to act as dressers and assistants generally. Those who follow the regiments to the field will establish themselves, each at a temporary depot, at such a distance or situation in the rear of his regiment as will insure safety to the wounded, where they will give such aid as is immediately required; and they are here reminded that whilst no personal consideration should interfere with their duty to the wounded, the grave responsibilities resting upon them render any unnecessary exposure improper.

19. The division surgeon will order to the hospital, as soon as it is located all the hospital supply wagons, hospital tents and furniture, and all the hospital stewards, cooks, and nurses belonging to the division. He will notify the officer commanding the division ambulance of the position of the hospital. When his duties permit, he will give his professional services at the hospital.

20. No medical officer will leave the position to which he has been assigned without permission; and any officer so doing is to be reported to the Medical Director of the corps, and to the Chief Medical Director.

21. Medical Directors of corps and division surgeons are required to have the following articles carried in the box of each ambulance, under the drivers seat:

| | | |
|---|---|---|
| Beef, extract, in 2-lb. tins, | lbs. | 6. |
| Buckets, leather, | No. | 1. |
| Kettles, camp, | No. | 1. |
| Lantern and candle, | No. | 1. |
| Spoons, table, | No. | 6. |
| Tumblers, tin, | No. | 6. |
| Hard bread, | lbs. | 10. |

The boxes will be kept locked. The surgeon-in-charge of the Brigade will keep the keys, and by weekly inspections ascertain that each ambulance has its full supply. In addition to the above, each ambulance is to be furnished with two litters, and one keg filled with water.

William A. Hammond,
*Surgeon-General U. S. Army.*

